# Supplementary material for: Limosilactobacillus reuteri promotes the expression and secretion of enteroendocrine- and enterocyte-derived hormones
Source: bioRxiv. 2024 Aug 31:2024.08.30.610555. Preprint. [Version 1] doi: 10.1101/2024.08.30.610555 (PMC11384013; doi:10.1101/2024.08.30.610555)
Supplement: Supplement 2 [file NIHPP2024.08.30.610555v1-supplement-2.pdf]

bioRxiv preprint doi: https://doi.org/10.1101/2024.08.30.610555; this version posted August 31, 2024. The copyright holder for this preprint (which was not certified by peer review) is the author/funder, who has granted bioRxiv a license to display the preprint in perpetuity. It is made available under aCC-BY 4.0 International license.

Supplemental Figure 1

Enterocyte markers

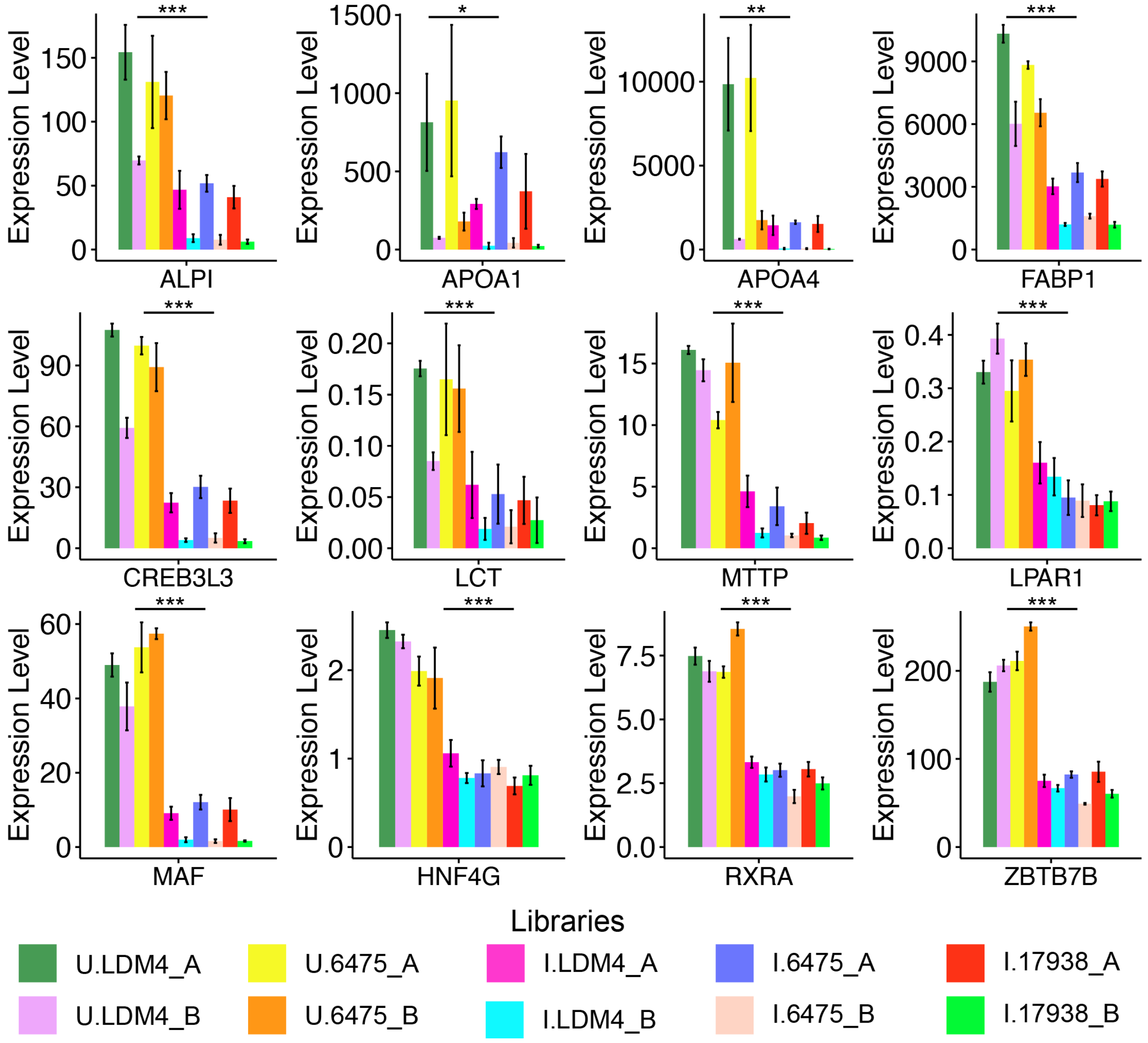

Supplemental Figure S1. Expression levels of enterocyte cell markers (*ALPI*, *APOA1*, *APOA4*, *FABP1*, *CREB3L3*, *LCT*, *MTTP*, *LPAR1*, *MAF*, *HNF4G*, *RXRA*, *ZBTB7B*) in uninduced and induced NGN3-HIOs. Libraries are labeled "U" for uninduced, "I" for induced" and "A" and "B" for the first and second batches of *NGN3*-HIOs. Expression levels shown are counts per million GeTMM transformed read counts. Significance of expression levels between the uninduced and induced libraries was calculated using a two-sample, two-sided, Mann-Whitney test. *, p<0.05, **, p<0.005, ***, p<0.0005.

bioRxiv preprint doi: https://doi.org/10.1101/2024.08.30.610555; this version posted August 31, 2024. The copyright holder for this preprint (which was not certified by peer review) is the author/funder, who has granted bioRxiv a license to display the preprint in perpetuity. It is made available under aCC-BY 4.0 International license.

Supplemental Figure 2

Enteroendocrine markers

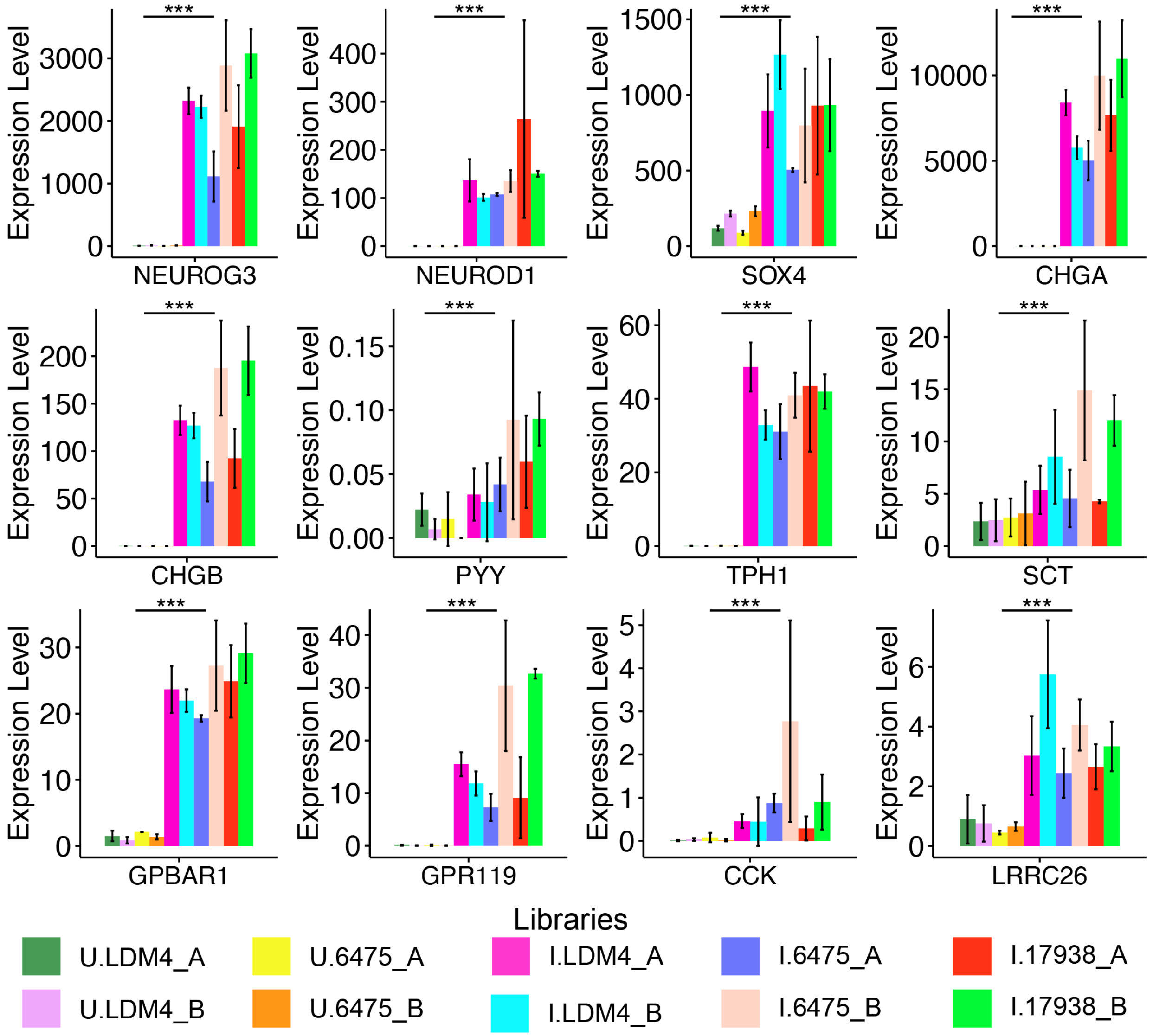

Supplemental Figure S2. Expression levels of enteroendocrine cell precursor markers (*NEUROG3*, *NEUROD1*, *SOX4*) and cell markers (*CHGA*, *CHGB*, *PYY*, *TPH1*, *SCT*, *GPBAR1*, *GPT119*, *CCK*, *LRRC26*) in uninduced and induced *NGN3*-HIOs. Libraries are labeled "U" for uninduced, "I" for induced" and "A" and "B" for the first and second batches of *NGN3*-HIOs. Expression levels shown are counts per million GeTMM transformed read counts. Significance of expression levels between the uninduced and induced libraries was calculated using a two-sample, two-sided, Mann-Whitney test. *, p<0.05, **, p<0.005, ***, p<0.0005.

bioRxiv preprint doi: https://doi.org/10.1101/2024.08.30.610555; this version posted August 31, 2024. The copyright holder for this preprint (which was not certified by peer review) is the author/funder, who has granted bioRxiv a license to display the preprint in perpetuity. It is made available under aCC-BY 4.0 International license.

Supplemental Figure 3

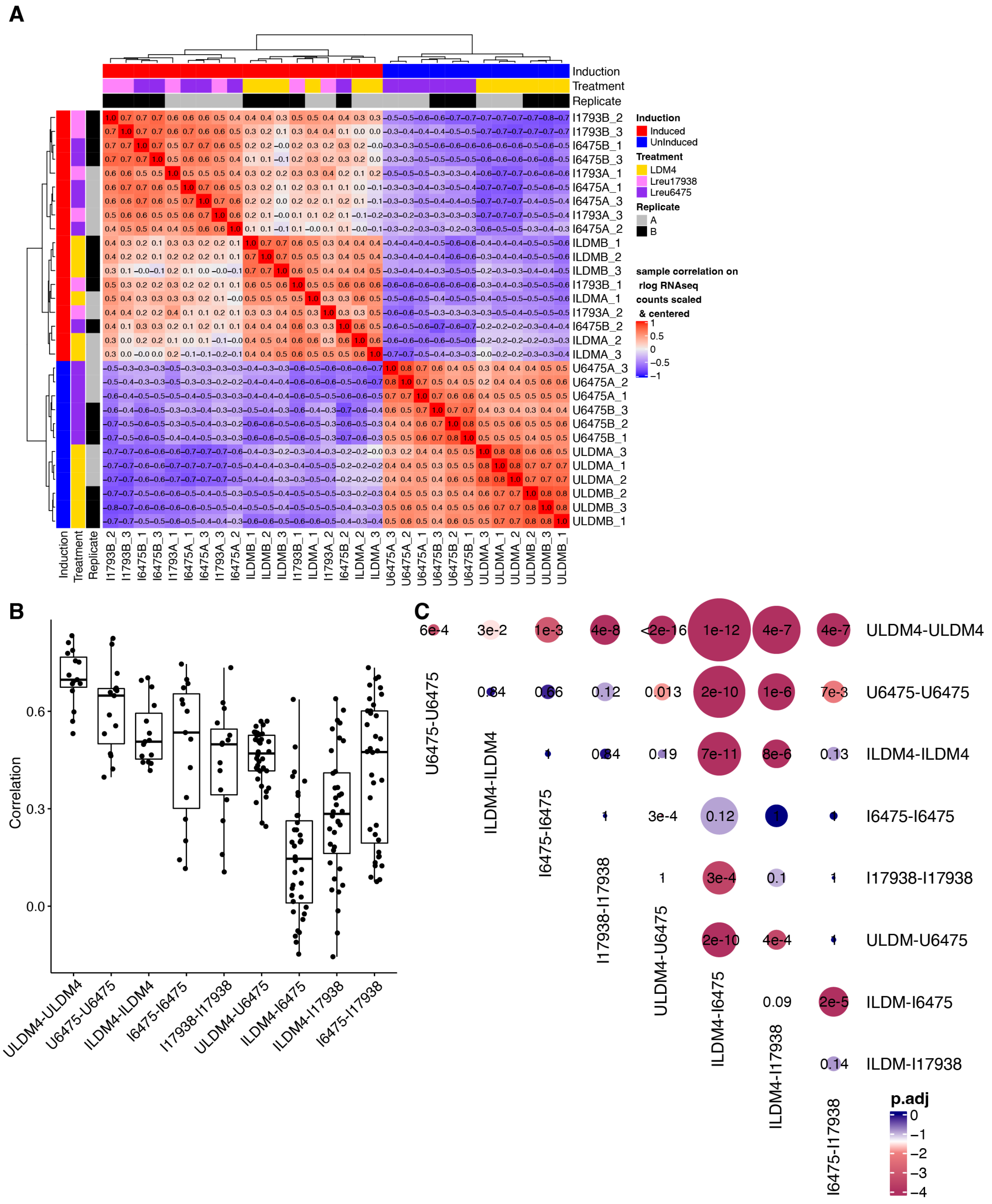

bioRxiv preprint doi: https://doi.org/10.1101/2024.08.30.610555; this version posted August 31, 2024. The copyright holder for this preprint (which was not certified by peer review) is the author/funder, who has granted bioRxiv a license to display the preprint in perpetuity. It is made available under aCC-BY 4.0 International license.

Supplemental Figure S3. Similarity and differences among *NGN3*-HIO transcriptomes. **A)** Boxplots of Pearson correlation values within and between transcriptomes. **B)** Correlogram of mean differences (circle size) and adjusted p-values (circle fill) between comparisons shown in A. Samples are listed, whereby “U” refers to uninduced *NGN3*-HIOs, “I” for induced *NGN3*-HIOs, “LDM4” for media only treatment, “6475” for *L. reuteri* 6475 treatment, “17938” for *L. reuteri* 17938 treatment, “A” or “B” refers to the biological replicate, and “1”, “2”, or “3” refers to the technical replicate within each biological replicate. **C)** Genes differentially regulated between *L. reuteri* 6475 and 17938 on induced *NGN3*-HIOs. The graph shows the $\log_2$ fold change expression of the gene for the indicated comparison. The bars are colored using the $\log_{10}$ scaled mean GeTMM counts to illustrate how abundantly expressed the gene is. Transparent overlays are used on genes not differentially expressed for the given comparison. Comparisons shown: U6475-ULDM4, *L. reuteri* 6475 on uninduced HIOs compared to LDM4 media control; I6475-ILDM4, *L. reuteri* 6475 on induced HIOs compared to LDM4 media control; I17938-ILDM4, *L. reuteri* 17938 on induced HIOs compared to LDM4 media control; I6475-I17938 *L. reuteri* 6475 compared to *L. reuteri* 17938 on induced HIOs; ILDM4-ULDM4, LDM4 media control on induced versus uninduced HIOs; I6475-U6475, *L. reuteri* 6475 on induced versus uninduced HIOs. For each, positive fold changes indicate genes upregulated by the condition listed first.

bioRxiv preprint doi: https://doi.org/10.1101/2024.08.30.610555; this version posted August 31, 2024. The copyright holder for this preprint (which was not certified by peer review) is the author/funder, who has granted bioRxiv a license to display the preprint in perpetuity. It is made available under aCC-BY 4.0 International license.

Supplemental Figure 4

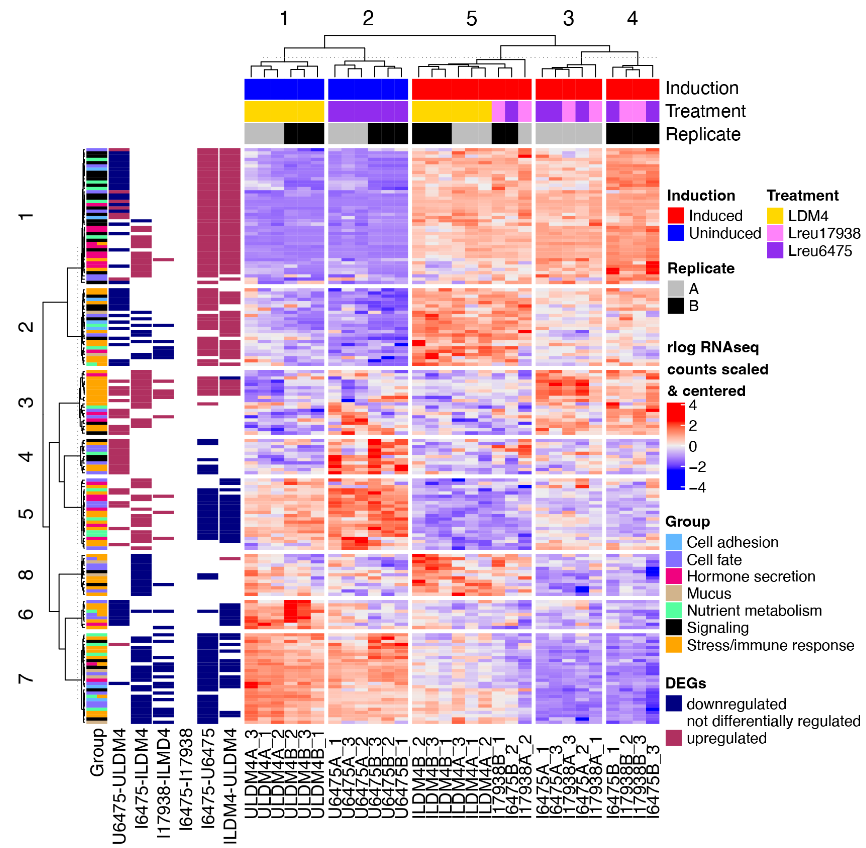

Supplemental Figure S4. Cluster analysis of DEGs belonging to functionally enriched groups. The heatmap shows gene expression values as rlog counts that were scaled and centered. Samples (the columns) along the bottom of the heatmap are labeled as "U" for uninduced *NGN3*-HIOs, "I" for induced *NGN3*-HIOs, "LDM4" for media only treatment, "6475" for *L. reuteri* 6475 treatment, "17938" for *L. reuteri* 17938 treatment, "A" or "B" for the biological replicate, and "1", "2", or "3" for the technical replicate within each biological replicate. Samples are annotated above the heatmap as shown in the legend. Genes (rows) were arranged by K-means clustering and annotated into groups as shown in the legend. For each sample comparison (e.g. U6475-ULDM4), if the gene was down or upregulated (e.g. higher in U6475 than ULDM4), a color is given as shown in the legend.

bioRxiv preprint doi: https://doi.org/10.1101/2024.08.30.610555; this version posted August 31, 2024. The copyright holder for this preprint (which was not certified by peer review) is the author/funder, who has granted bioRxiv a license to display the preprint in perpetuity. It is made available under aCC-BY 4.0 International license.

Supplemental Figure 5

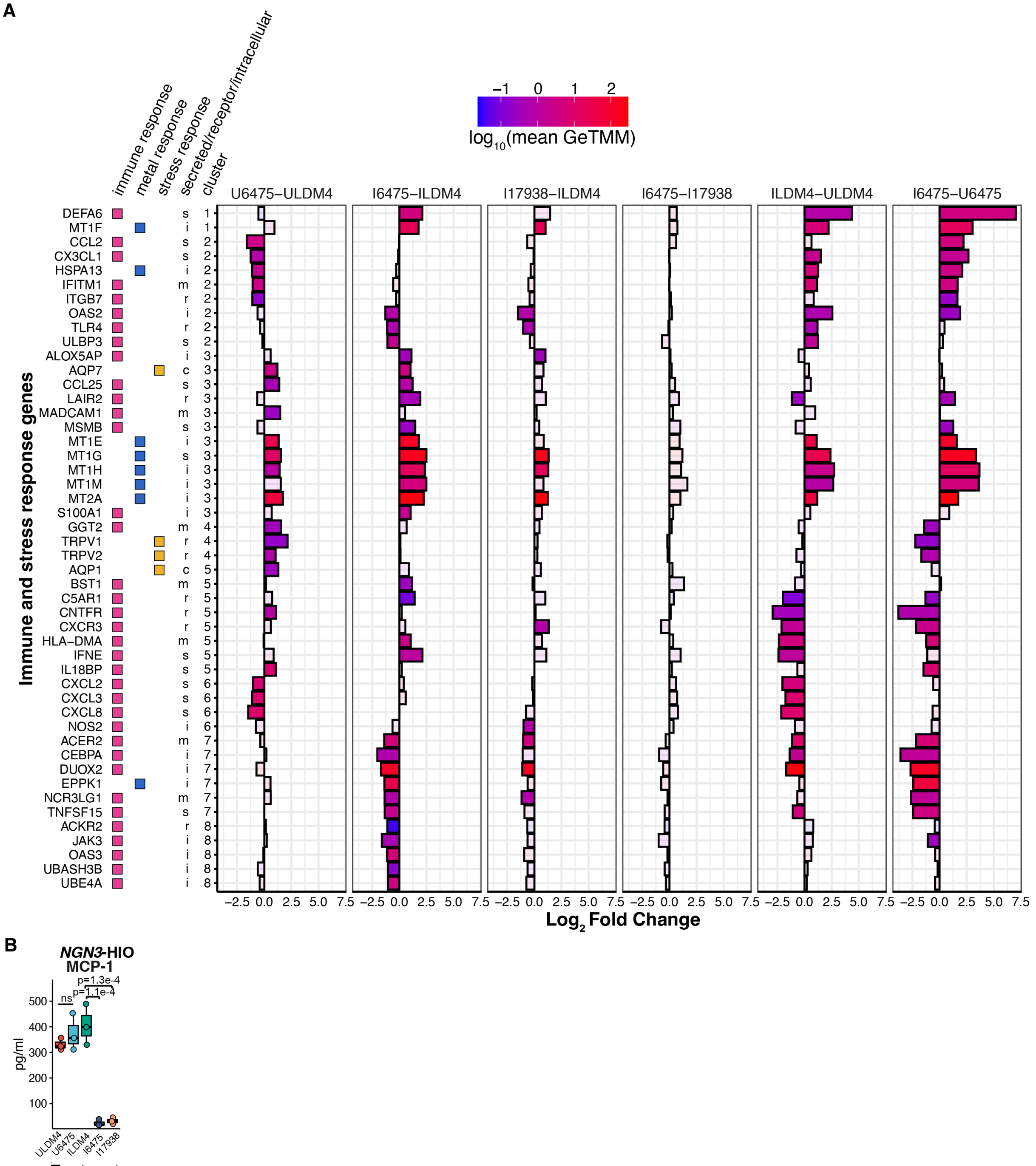

Supplemental Figure S5. *L. reuteri* regulates immune, metal, and stress response. **A)** Immune, metal, and stress genes differentially regulated by *L. reuteri*. The genes are annotated with their function, whether they are secreted, a receptor, or intercellular, and what cluster they belong to relative to Supplemental Figure S4. The graph shows the log_2 fold change expression of the gene for the indicated comparison. The bars are colored using the log_{10} scaled mean GeTMM counts to illustrate how abundantly expressed the gene is. Transparent overlays are used on genes not differentially expressed for the given comparison. Comparisons shown: U6475-ULDM4, *L. reuteri* 6475 on uninduced HIOs

bioRxiv preprint doi: https://doi.org/10.1101/2024.08.30.610555; this version posted August 31, 2024. The copyright holder for this preprint (which was not certified by peer review) is the author/funder, who has granted bioRxiv a license to display the preprint in perpetuity. It is made available under aCC-BY 4.0 International license.

compared to LDM4 media control; I6475-ILDM4, *L. reuteri* 6475 on induced HIOs compared to LDM4 media control; I17938-ILDM4, *L. reuteri* 17938 on induced HIOs compared to LDM4 media control; I6475-I17938 *L. reuteri* 6475 compared to *L. reuteri* 17938 on induced HIOs; ILDM4-ULDM4, LDM4 media control on induced versus uninduced HIOs; I6475-U6475, *L. reuteri* 6475 on induced versus uninduced HIOs. For each, positive fold changes indicate genes upregulated by the condition listed first. **B)** MCP-1 protein levels measured by Luminex on uninduced (U) or induced (I) HIOs treated with *L. reuteri* 6475 or 17938.

bioRxiv preprint doi: https://doi.org/10.1101/2024.08.30.610555; this version posted August 31, 2024. The copyright holder for this preprint (which was not certified by peer review) is the author/funder, who has granted bioRxiv a license to display the preprint in perpetuity. It is made available under aCC-BY 4.0 International license.

Supplemental Figure 6

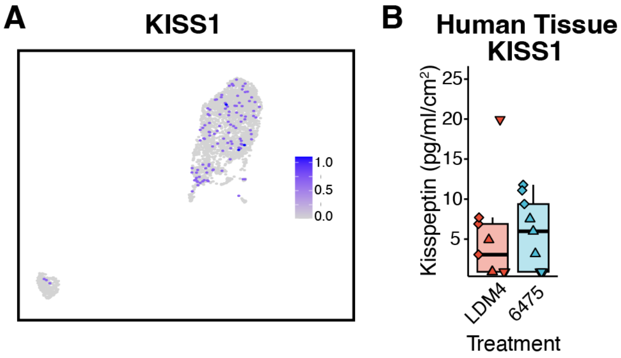

Supplemental Figure S6: KISS1 may be produced in the intestinal epithelium. **A)** UMAP of KISS1 using the Gut Cell Atlas adult jejunum data. **B)** Lack of secretion of kisspeptin in response to bacterial media control (LDM4) and *L. reuteri* 6475 conditioned media from *ex vivo* human jejunal intestinal tissue. Shape represents unique human intestinal donors. Significance was determined using a linear mixed model with $p<0.05$ considered as significant.

Supplemental Tables available in Excel document

Supplemental Table 1: Sequencing reads per library. Number of sequencing reads for each sample after filtering and aligning to the reference human genome (see Methods).

Supplemental Table 2: Genes differentially regulated between *L. reuteri* strains 6475 and 17938 in induced and uninduced HIEs. Libraries are labeled “U” for uninduced, “I” for induced” and “A” and “B” for the first and second batches of organoids. For each comparison column, e.g. U6475-ULDM, “0” means no difference, “-1” means ULDM has higher expression values than U6475, “1” means U6475 has higher expression values than ULDM. Expression levels shown are computed using the rlog. Output from DESeq2 (base mean (average of count values post normalization for size factors), log2 fold change, log2 fold change standard error, test statistic from a Wald test, p-value, and adjusted p-value using the Benjamini-Hochberg procedure) for each comparison are given.

Supplemental Table 3: Enriched functional groups in *L. reuteri* over media alone DEGs. Annotations were taken from the indicated annotation group as annotated by the PANTHER classification system and Reactome annotated pathways. Groupings were manually assigned with the intention of generalizing the types of functional groups among the data. Enriched refers to whether the functional group is enriched in the set of DEGs or depleted. DEG Up-regulated (+) or Down-regulated (-) displays if the genes within the functional group were up or down-regulated by the respective *L. reuteri* strain compared to the media alone control.

Supplemental Table 4: Functional DEGs in *L. reuteri* over media alone annotations. DEGs belonging to a functional group are annotated at three levels, upper, middle, and final, with increasing levels of resolution. As well, the DEGs are classified by a subtype giving information about their cellular location. DEG up- or downregulation information, gene information, and output from DESeq2 are given as in Supplemental Table 2. GeTMM transformed read counts are given as well.
